# Gangrene of the Mouth in Children

**Published:** 1849-07

**Authors:** 


### GANGRENE OF THE MOUTH IN CHILDREN.

Dr. Hy. Hunt prescribes *chlorate of Potash* internally, in all cases of the above description, according to the age of his patients, in doses of a scruple to a drachm, dissolved in two ounces and a half or three ounces of water, conveniently sweetened. Of this solution the little patients take half a tea spoonful every hour.

The good results of this medicine are not long in manifesting themselves, a percep[illegible]e amelioration being observed after the second, or, at the latest, the third day. The fetid odor promptly diminishes, the saliva flows less abundantly, the ulcerations rapidly assume a marked tendency to cicatrization, and the cure is complete at the end of a week. It is right to remark that the progress is not quite so rapid if there exists a slough, but even in this case the slough is seen to detach itself more readily than usual, and to be replaced by fleshy granulations of a healthy aspect.

This treatment, according to the author, has never failed in his hands, unless the children had already arrived at the last degree of exhaustion before being submitted to it; and he then adds that the advantages of this preparation are confirmed by sixteen years' experience.

It is to be regretted, that after obtaining so much success, Dr. Hunt should have confined himself to citing four cases, one of which terminated in death. But in this latter case he was called in, he says, at the last stage of the disorder, when the gangrene had attacked the entire cheek; nevertheless, even in this subject he was able to ascertain that the remedy, although applied too late to conquer the complaint, had hastened the fall of the sloughs. To the chlorate of potash, joins the use of a laxative, composed as follows—

Hydrarg. submur. gr. j.
Pulv. Rhei. gr. viij.
Potass. sulph. gr. x.

It is scarcely probable that Dr. Hunt has obtained the success for which he gives credit to the chlorate of potash, in cases where he has had to do with gangrenous aphthæ only, and that in those cases where this medicine failed, the gangrene was of that nature which attacks the cheek throughout its thickness. However this may be, the method recommended, merits, undoubtedly, the attention of practitioners, and a trial on all proper occasions.

